# Genetic Analysis of 27 Y-STR Haplotypes in 11 Iranian Ethnic Groups

**DOI:** 10.34172/aim.2024.13

**Published:** 2024-02-01

**Authors:** Somayeh Alinaghi, Marzieh Mohseni, Zohreh Fattahi, Maryam Beheshtian, Fatemeh Ghodratpour, Farzane Zare Ashrafi, Sanaz Arzhangi, Khadijeh Jalalvand, Reza Najafipour, Hamid Reza Khorram Khorshid, Kimia Kahrizi, Hossein Najmabadi

**Affiliations:** ^1^Genetics Research Center, University of Social Welfare and Rehabilitation Sciences, Tehran, Iran

**Keywords:** Ethnic group, Haplotype, Iran, Y-STR, Yfiler® Plus

## Abstract

**Background:**

The study of Y-chromosomal variations provides valuable insights into male susceptibility in certain diseases like cardiovascular disease (CVD). In this study, we analyzed paternal lineage in different Iranian ethnic groups, not only to identify developing medical etiology, but also to pave the way for gender-specific targeted strategies and personalized medicine in medical genetic research studies.

**Methods:**

The diversity of eleven Iranian ethnic groups was studied using 27 Y-chromosomal short tandem repeat (Y-STR) haplotypes from Y-filer® Plus kit. Analysis of molecular variance (AMOVA) based on pair-wise R_ST_ along with multidimensional scaling (MDS) calculation and Network phylogenic analysis was employed to quantify the differences between 503 unrelated individuals from each ethnicity.

**Results:**

Results from AMOVA calculation confirmed that Gilaks and Azeris showed the largest genetic distance (R_ST_=0.35434); however, Sistanis and Lurs had the smallest considerable genetic distance (R_ST_=0.00483) compared to other ethnicities. Although Azeris had a considerable distance from other ethnicities, they were still close to Turkmens. MDS analysis of ethnic groups gave the indication of lack of similarity between different ethnicities. Besides, network phylogenic analysis demonstrated insignificant clustering between samples.

**Conclusion:**

The AMOVA analysis results explain that the close distance of Azeris and Turkmens may be the effect of male-dominant expansions across Central Asia that contributed to historical and demographics of populations in the region. Insignificant differences in network analysis could be the consequence of high mutation events that happened in the Y-STR regions over the years. Considering the ethnic group affiliations in medical research, our results provided an understanding and characterization of Iranian male population for future medical and population genetics studies.

## Introduction

 The pathophysiology of several diseases, including cancer, neuro-developmental, neuro-degenerative, and cardiovascular diseases (CVDs), is influenced by sex, affecting male development and metabolic homeostasis.^1-3^ Numerous studies have reported how Y-chromosome haplotypes and haplogroups may help in understanding the etiology of complex diseases or manifestation of a medical outcome.^4-6^ Recently published studies have shown that the Y-chromosome can play a role in CVDs and hypertension manifestation.^7-9^ Males with haplogroup I of the Y-chromosome would have 50% higher age-adjusted risk of CVDs than other Y chromosome lineages. Besides, transcriptome molecular pathway in males with haplogroup I revealed significant differential expression between genes related to inflammation and immunity. Furthermore, some of these genes are relevant to atherosclerosis.^10^ Consequently, the paternal lineage may inform about the increased risk of developing heart disease and the immune system’s response, affecting coronary artery disease (CAD) occurrence.^10,11^ For example, the *UTY* and *KDM5D* genes modulate the inflammatory and adaptive immunity processes that contribute to atherosclerosis and the resulting outcomes of CVD.^2,12,13^ Mutations in the *SLY* and *RBMY* genes impact experimental allergic encephalomyelitis (EAE) and experimental myocarditis.^14^ Autism and intellectual disabilities are associated with *NLGN4Y*.^15^ Additionally, male breast and prostate cancer manifest through deletion in the putative *TMSB4Y* and *TSPY2* tumor suppressor genes on the Y-chromosome.^16,17^ Exploring paternal lineage with Y-chromosomal short tandem repeat (Y-STR) markers provides a unique lens into the inheritance of sex-related medical outcomes, and offering valuable insights into disease risk assessment, personalized treatment plans, and advancing precision medicine initiatives.

 Iran’s population is a combination of various ethnic and cultural groups. A genetically diverse population was established in the area by a mixture of several linguistic communities with differing claims to common ancestry.^18-21^ Regarding gene flow, the Iranian plateau has been limited by topological barriers like the Zagros and Alborz mountains, the Caspian Sea, the Dasht-e Kavir and the Dash-e Lut deserts, and the Persian Gulf.^22,23^ Furthermore, as a transcontinental route, Iran was influenced by past migration and expansion events.^24^ With the emergence of modern humans out of Africa, Iran’s territory played a pivotal role in pre-historic and historic migratory events between Africa, Asia, and Europe.^25^ Over the centuries, the Iranian plateau was the destination for male-dominant directional expansion of Indo-European (IE) speakers from Europe, Arab Muslims from West Asia and North Africa (in the 7th century), and Turkic speakers from central Asia (in the 13th century).^26-28^ Investigation of sex-specific population patterns in paternal and maternal lineages would be ideal for determining potentially different degrees of genetic diversity and migration patterns.^29^ Male population stratification assessment among single worldwide populations and between continental groups can be determined based on Y-STR markers using single markers and haplotype frequency distributions.^30^ Y-chromosomal markers provide exciting insights into the past demographic events of a population, such as male-specific migration, especially since the Industrial Revolution, which blurs the picture of historical population structure and admixture events.^31^ Different studies have shown that Y-STR haplotype spectra are different in Western Asia compared with other geographical regions in the world, which is proposed to be a corridor for ancient human migrations.^32,33^

 In the past few years, a larger number of studies have investigated Y-STR data from countries and regions in vicinity of Iran and beyond. This includes studies on Armenia/Turkey/Caucasus/Georgia,^34-36^ Afghanistan,^37^ and Arab countries,^32,38-40^ as well as Russia,^41^ whose southern part is considered to have hosted the speakers of Proto-Indo-European (PIE), Pakistan,^42^ India,^43^ Greece,^44^ the Levant,^45^ Central Asian Uyghurs^46^ and East and West Africans.^47,48^ Nevertheless, Y-STR data from Iran are sparse, both with respect to the geographical and ethnical coverage. A study from 2009 compiled Y-filer data of 259 males from six ethnic groups in Iran and Azerbaijan,^49^ complemented by another 259 Y-filer samples from the East and Southeast of Iran.^50^ More Y-filer samples were added from Mazandaran, Gilan (n = 209)^51^ and Golestan (n = 106) provinces.^52^ However, these studies neither cover large parts of Iran, nor was their sampling always strictly based on ethnic groups, but rather geography, with limited number of studied markers set. Therefore, they are of limited use in investigating the male population structure in Iran and inferring past migration events. Higher mutation rates of STRs can lead to a high diversity of haplotypes that share a recent ancestor. As frequency distributions of Y-STR haplotypes are applicable for studying genetic population differences, their profiles give a more accurate insight into the paternal history of mixed populations.^53,54^

 A genome-wide genotyping for 1021 DNA samples from eleven Iranian ethnicities demonstrated that the CIC (Central Iranian Cluster) comprises seven ethnic groups that show a large genetic overlap, and the four remaining ethnic groups show different ancestral populations.^55^ In this regard, investigation of uniparental markers such as Y-chromosomal STR haplotypes in the Iranian population could establish the role of Y-chromosome in identifying group’s susceptibility for developing health problems.^56^ This genetic information can advise assessing variations in medical approaches. Previous studies on Iranian male samples demonstrated that different migration events and critical geographic barriers led mainly to large and significant diversities between Iranian samples.^21,49,52,57^ To adjust the interpretable findings of genetic variability of Y-STRs, we performed an adequate design and analysis of Y-STR markers and haplotypes in the Iranian population as an interpretation for future population and medical genetics studies like risk assessment, treatment strategies and prevention measures.

## Materials and Methods

###  Iranian Samples

 To collect data on age, sex, ethnicity, demographics, and health status, written informed consent was obtained from all participants prior to the study, following the rules of the Research Ethics Committee, University of Social Welfare and Rehabilitation Sciences (USWR), Tehran, Iran. Approval to undertake the work was obtained from USWR Research Ethics Committee (Approval number IR.USWR.REC.1395.376 and IR.USWR.REC.1400.136).

 In the current study, we included 530 unrelated healthy males from the Iranome project samples (http://www.iranome.ir)^58^ (49.57% of total samples) representing eleven Iranian ethnic groups, namely Iranian Arabs, Iranian Azeris, Iranian Baluchis, Iranian Kurds, Iranian Lurs, Iranian Gilaks, Iranian Mazanderanis, Iranian Sistanis, Iranian Persians, Iranian Turkmens, and Iranian Persian Gulf Islanders (PGI). The concentration of all DNA samples to use in STR analysis was quantified before application. Highly degraded samples and samples containing insufficient DNA were excluded from STR typing as they cannot be retrieved with capillary electrophoresis. A total number of 503 male DNA samples were selected for the subsequent procedures (Table 1). Previously in the Iranome Project, individuals who met the inclusion criteria were collected from different provinces of Iran.^58^ The individuals were assigned to each ethnic group if they originated from the same ethnic background for at least two generations, whereas close relatives and individuals from mixed ethnic groups were excluded. Besides, individuals in each ethnic group were above 40 years of age at the time of admission to reduce the possibility of genetic diseases developing later in life. In general, the average age of all ethnic group’s participants was 45 years, and sampling was performed based on total population, equally from males and females. In the final stage, a clinician evaluated all the participants of the project.

**Table 1:** Samples Filtration and Quality Control

**Ethnic Groups**	**Samples Before QC**	**Samples After QC**
Iranian Arabs	52	50
Iranian Azeris	51	50
Iranian Baluchis	52	50
Iranian Gilaks	50	48
Iranian Kurds	42	36
Iranian Lurs	42	41
Iranian Mazanderanis	50	46
Iranian Persian Gulf Islanders	51	48
Iranian Persians	40	36
Iranian Sistanis	50	49
Iranian Turkmens	50	49
Total	530	503

QC, quality control.

###  Quality Control

 All experimental procedures were operated entirely according to Yfiler® Plus kit controls (Cat. No. 4484678 and 4482730) and internal laboratory standards. To evaluate the efficiency of the amplification steps, a panel of standards was used for PCR amplification, PCR product sizing, and genotyping which were: Yfiler® Plus DNA Control 007, GeneScan^TM^–600 LIZ^TM^ Size Standard v2.0 (Cat. No. 4408399) (Thermo Fisher Scientific’s Yfiler® Plus PCR Amplification Kit, Life Technologies Ltd, Woolston, United Kingdom), Yfiler® Plus Allelic Ladder. Additional purifications were performed before proceeding to STR typing to obtain required conditions that produced optimum PCR allele typing results and appropriate cycle number and avoid off-scale peaks.

 The kit developed optimal performance to improve the resolution of paternal lineages and discriminate between closely related males based on the updated and revised guidelines from the Scientific Working Group on DNA Analysis Methods (SWGDAM, December 2012 and March 2022). The kit’s sensitivity and reliability were provided based on comparable studies, ensuring a robust foundation for accurate and consistent outcomes.^59-61^ This paper follows the guidelines for the publication of population data proposed by the journal.

###  Y-STR Typing

 DNA extraction was performed on blood samples following the standard salting out procedure.^62^ Samples were typed for all 27 Y-STR markers of the Y-filer Plus kit using the manufacturer’s protocol (Thermo Fisher Scientific’s Y-filer® Plus PCR Amplification Kit, Life Technologies Ltd, Woolston, United Kingdom). Although Duplicated markers DYS385a/b and DYS387S1a/b were typed in all samples, the representative genotypes were discarded in all subsequent analyses due to allele assignment disruption. This kit examined ten significant loci; three loci were highly polymorphic (DYS460, DYS481, and DYS533), and seven loci (DYS387S1a/b, DYS449, DYS518, DYS570, DYS576, DYS627) were rapidly mutating (RM). The remaining 17 markers were referred to as the ‘large marker set’ (DYS389I/II, DYS635, DYS458, DYS19, YGATAH4, DYS448, DYS390, DYS391, DYS456, DYS438, DYS392, DYS437, DYS393, DYS439, DYS385 a/b). The combination of RM Y-STRs with an increased number of targeted loci has empowered discrimination in high levels of population haplotype diversity in the Y-filer Plus kit.^59^ Amplified PCR products were produced in approximately 30 cycles of PCR on a Veriti® 96-Well Thermal Cycle, along with negative and positive controls, to optimize thermal cycling conditions. PCR product separation and detection were performed on an ABI 3500xL Genetic Analyzer machine (Thermo Fisher Scientific^TM^, Massachusetts, United States) filled with POP-6^TM^ polymer. Allele values were assigned using the reference ladder provided with each kit and repeat numbers indicated by the GeneMapper® ID-X v1.6 software (Thermo Fisher Scientific, Massachusetts, United States). In this study, the number of repetitions in DYS389I was deducted from DYS389II, referring to the repeat numbers at individual loci rather than the repeat numbers revealed by the multiplex genotyping method. A total of 403 samples (80.12%) were typed for all 27 Y-STR markers of the Y-filer Plus kit using the manufacturer’s protocol. The remaining 19.88% (100 samples) were, with insight, excluded from complete retyping, as the data from the ‘large marker set’, comprising key markers in the Y-chromosome haplotype, had been previously obtained and considered to be sufficient for robust statistical analysis. This strategic approach ensures both efficiency in resource utilization and methodological velocity in the desire for meaningful results.

###  Statistical Analysis

####  Analysis of Molecular Variance 

 Analysis of population variation between eleven Iranian subpopulations was quantified based upon pair-wise sum of squared size difference (R_ST_). In the analysis of molecular variance (AMOVA) calculation, for significance testing *P *values of R_ST_ > 0 and using randomization with 1000 permutations, we employed Arlequin v 3.5.2.2.^63^ The DYS385a/b and DYS387S1a/b markers were not included in the AMOVA because they do not allow for easy calculation.

####  Multidimensional Scaling Analysis

 Multidimensional scaling (MDS) was utilized to visualize the average distances in Y-STR genetic variation between ethnic groups. It was based upon pair-wise R_ST_ between sampling sites as estimated with Arlequin (see above) and was implemented in the *cmdscale* function of R v4.1.3.^64^ MDS was used to investigate genetic similarities between populations.^65^ Plots of the first two MDS components, C1 and C2, capturing the most and second most R_ST_-defined variation were generated with R using in-house scripts. For R_ST_ calculation and MDS analysis, multi-locus markers, haplotypes containing duplications, along with null alleles and micro-variants, were disregarded from the analysis of the respective marker set.

###  Median-Joining Network Analysis

 To identify phylogenetic connections between samples containing unique and uncommon variations based on Y-STR haplotypes, we used the stepwise star-contraction, Median-Joining (MJ), and Maximum-Parsimony (MP) algorithm calculations with NETWORK v10 and NETWORK Publisher v2.1.2.5 as described on the Fluxus Engineering website (Fluxus Technology Ltd, London, United Kingdom) (https://www.fluxus-engineering.com).^66^ All Y-STR loci’s weights were adjusted based on the marker-specific mutation rate (https://yhrd.org/pages/resources/mutation_rates). In network construction, each marker was weighted by the inverse of its marker-specific mutation rate and these rates were used for calibration. Then, the ages of nodes within the network can be estimated. In the network phylogenic analysis, the deleted alleles were coded ‘99’ in input files and thereby considered as missing data. Additionally, duplicated loci (DYS385a/b; DYS387S1a/b) were removed for network construction, as it is not possible to associate particular alleles with specific copies. We applied the star-contraction, MJ, and MP options of the NETWORK software in our analyses.

## Results

###  Sample Characteristic 

 The 503 male samples originated from eleven Iranian ethnic groups, ranging from 36 to 50 per group (Table 1). A number of 435 unique haplotypes were derived from 503 individuals using the 27 Y-STRs Y-filer® Plus kit. There were 34 haplotypes shared among two individuals.

###  AMOVA and MDS Analysis

**Figure 1:**
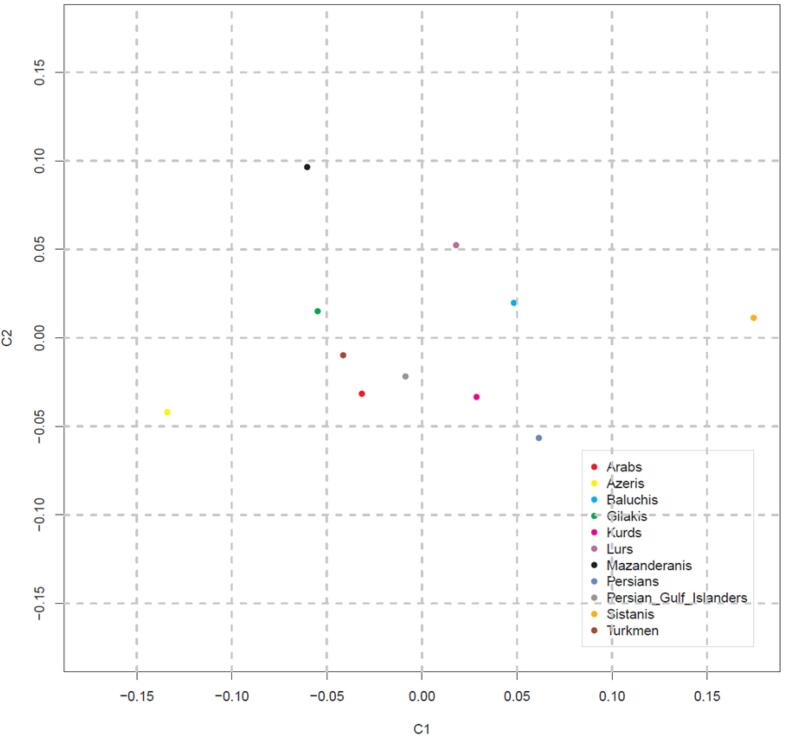
Multidimensional Scaling (MDS) Analysis of 11 Iranian Ethnic Groups Based on Pairwise Genetic Distances (RST). MDS plot shows the relationships between all iranian male individuals

 In population distance analysis, all pair-wise comparisons resulted in R_ST_ values which were significantly different from zero (*P* < 0.001) and genetic distance (R_ST_) values for pair-wise comparisons of haplotypes between ethnic groups were further explored (Table 2). The results indicated that Gilak and Azeri ethnicities are located at the largest genetic distance (R_ST_ = 0.35434) in comparison with other ethnic groups. In contrast, the least considerable genetic distances are seen in Sistanis and Lurs (R_ST_ = 0.00483) and Arabs and Lurs (R_ST_ = 0.00521). The largest R_ST_ values were observed for Azeris in comparison with other ethnic groups, which shows the considerable difference of Azeris with other ethnicities. Meanwhile, in the value of R_ST_, among the other ethnic groups, the Turkmen ethnicity demonstrated the least distance from Azeris (R_ST_ = 0.13284). The degree and significance of differentiation between groups were assessed by AMOVA. The AMOVA results demonstrated that the Y-STR haplotypes differ significantly across ethnic groups; 11.57% of the genetic variance reflects differences among population groups, whereas 88.43% reflects differences within ethnic groups, with a Fixation Index (F_ST_) of 0.11574 (Table 3). The MDS analysis compared all eleven ethnic groups and was performed using R_ST_ values (Figure 1). Ultimately, MDS analysis of pair-wise R_ST _from eleven Iranian ethnic groups revealed the absence of resemblance between distinct Iranian ethnicities.

**Table 2:** R_ST_ Values Between Pairs of Ethnic Groups

	**Iranian Arabs**	**Iranian Azeris**	**Iranian Baluchis**	**Iranian Gilaks**	**Iranian Kurds**	**Iranian Lurs**	**Iranian Mazanderanis**	**Iranian Persian Gulf Islanders**	**Iranian Persians**	**Iranian Sistanis**	**Iranian Turkmens**
Iranian Arabs	0	0.23142	0.03069	0.06957	0.11522	0.00521	0.03895	0.08141	0.06524	0.04776	0.01662
Iranian Azeris	0.23142	0	0.27394	0.35434	0.15842	0.24810	0.18979	0.18743	0.28124	0.22494	0.13284
Iranian Baluchis	0.03069	0.27394	0	0.09219	0.10617	0.03040	0.06570	0.08968	0.09231	0.06049	0.07288
Iranian Gilaks	0.06957	0.35434	0.09219	0	0.18996	0.09102	0.16729	0.19571	0.13030	0.10057	0.12947
Iranian Kurds	0.11522	0.15842	0.10617	0.18996	0	0.08427	0.06935	0.06093	0.14353	0.06422	0.08559
Iranian Lurs	0.00521	0.24810	0.03040	0.09102	0.08427	0	0.01618	0.07674	0.03898	0.00483	0.02712
Iranian Mazanderanis	0.03895	0.18979	0.06570	0.16729	0.06935	0.01618	0	0.08485	0.10991	0.02458	0.02600
Iranian Persian Gulf Islanders	0.08141	0.18743	0.08968	0.19571	0.06093	0.07674	0.08485	0	0.07213	0.08535	0.08374
Iranian Persians	0.06524	0.28124	0.09231	0.13030	0.14353	0.03898	0.10991	0.07213	0	0.06267	0.08639
Iranian Sistanis	0.04776	0.22494	0.06049	0.10057	0.06422	0.00483	0.02458	0.08535	0.06267	0	0.04099
Iranian Turkmens	0.01662	0.13284	0.07288	0.12947	0.08559	0.02712	0.02600	0.08374	0.08639	0.04099	0

**Table 3:** Analysis of Molecular Variance (AMOVA) Results

**Source of Variation **	***df ***	**Sum of Squares**	**Variance Components**	**Percentage of Variation (%)**	***P*** ** Value**
Among populations (Va)	10	2327.729	4.36654	11.57	< 0.001
Within populations (Vb)	492	16414.096	33.36198	88.43	< 0.001
Total	502	18741.825	37.72852	100	
Fixation Index	F_ST_: 0.11574				

Va, Variance for among populations; Vb, Variance for within population; df, degrees of freedom. Significance of tests was based on 1023 permutations.

###  Y-STR Network Analysis 

**Figure 2:**
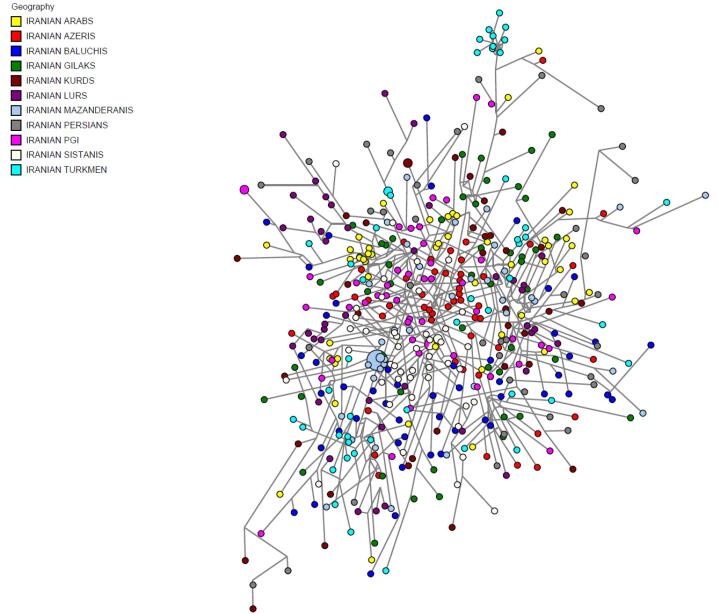
Median-Joining Haplotype Network for 27 Y-STR for 11 Iranian Ethnic Groups

 To reveal the detailed structures of Iranian male populations, a median-joining network using Y-STRs was constructed, derived from the data on all markers with mutation rates under 0.0035 (Table 4). Within both MJ and MP, no portioning of populations was observed by ethnic groups, and all male individuals were distributed throughout the network. No phylogenic clustering of ethnic groups within network analysis became apparent (Figure 2). It is evident that in the network results overall, many mutation events occurred between the samples, which indicates that Iranian male samples do not have a specific clustering structure. Absence of substructure between samples could be the result of high mutation events that happened in the Y-STR regions during past years.

**Table 4:** Y-STR Mutation Rates

**Mutation **	**Rate**
DYS19	2.1e-03
DYS389I	2.39e-03
DYS389II	4.60e-03
DYS390	2.0e-03
DYS391	2.4e-03
DYS392	0.52e-03
DYS393	1.2e-03
DYS437	1.3e-03
DYS438	0.35e-03
DYS439	5.1e-03
DYS448	1.4e-03
DYS456	4.3e-03
DYS458	6.6e-03
DYS635	4.2e-03
YGATAH4	2.5e-03
DYS576	12.1e-03
DYS481	4.6e-03
DYS533	3.0e-03
DYS570	9.2e-03
DYS627	14.7e-03
DYS460	4.4e-03
DYS518	11.9e-03
DYS449	1.02e-03

Source: https://yhrd.org/pages/resources/mutation_rates Since it is not possible to associate particular alleles with particular copies of duplicated loci (DYS385a/b; DYS387S1a/b), the mutation rates of these loci were not included.

## Discussion

 In the largest male genetic study of the Iranian population to date, we investigated the pattern of Y-chromosome STR markers in 503 samples from eleven different Iranian ethnic groups. The sampling sites were chosen to represent different ethnic groups. Our study revealed that regarding the Y-STRs, Iranian ethnic groups show largely overlapping genetic variations with no or only subtle substructure (*P* > 0.05).

 The overall results from AMOVA calculations indicate that the Azeris ethnic group showed high R_ST_ values among all the studied sub-populations; meanwhile, among Azeris ethnic group allocated R_ST_ values, the Turkmen ethnicity has the lowest value comparing other ethnic groups. In a previous study on 1021 Iranian samples,^55^ the authors demonstrated that the Iranian population comprises a group of seven ethnicities (CIC) among other groups with largely overlapping autosomal variations, including Iranian Azeris. Meanwhile, the remaining four ethnic groups that showed large degrees of distance were Iranian Baluchis, Iranian Sistanis, Iranian Turkmens, and Iranian inhabitants of the islands in Persian Gulf. Notably, in terms of paternal Y-STR markers, the Azeri ethnicity, which with respect to autosomal data belongs to CIC, has a considerable distance from other ethnicities but is still close to Turkmens. Based on our results of Y-chromosome haplotypes, the Iranian population has a different structure in Y-STR markers compared to autosomal variants. In this area, if we could assume Y-STR markers as representatives of disease-causing genetic variants on Y-chromosome, we would expect the carrier frequencies of Y-chromosome variants to be approximately the same for all ethnic groups, excluding the Azeris.

 During the 6thcentury, the Turkic peoples of Central Asia gradually spread westward; they were dominating male warriors that often took local women.^67^ These expansions eventually played a significant role in shaping the demographic features of the inhabitants.^68^ Our observations highlight this possibility that the Azeri and Turkmen ethnicities have been influenced by male-dominant expansions across Central Asia over the past thousand years, leading to the spread of their genes through the male lineage. To our knowledge, based on their earliest origins in Central Asia to their present-day genetic and linguistic diversity, these two ethnicities have the same linguistic history. These migrations of Turkic speakers and interactions with other genetic ancestries had a profound influence on the development of the Turkic languages, leading to the emergence of numerous dialects and subgroups within the language family. Therefore, the genetic admixture of people in the North West and East of Iran, i.e. the Azeri and Turkmen ethnicities, might have originated from Turkic-speaking genetic ancestries.^69,70^ On the other hand, the gradual influx of westward male and female migrations in the East Eurasia could be the reason that the Turkmen ethnic group is genetically close to Azeris but does not have significantly large genetic differences from other ethnicities,^71,72^ which could be considered in medical genetics applications.^73,74^

 The results of network analysis demonstrated a large variability with subtle substructure of Iranian male Y-STR marker variation. STR loci on Y-chromosome can exhibit high complex repeat motif variability and a unique inheritance pattern due to lack of recombination and the evolutionary mechanisms driving the increased mutation rates at these markers.^75,76^ The high level of mutation rates between samples in the Network analysis results confirmed the same genetic structure result for the different ethnic groups of the Iranian male population. The results indicate high variability with subtle substructure of Y-STR marker variation, which could be the cause of unique inheritance pattern and high mutation rates at these markers.^77^ Y-STR markers are passed down from father to son in a lineage-specific manner, which means that each lineage may have different patterns of Y-STR marker alleles. Additionally, the Y chromosome is not subject to recombination events like the other chromosomes, which means that mutations in Y-STR markers can accumulate over generations without being shuffled or diluted by recombination events.^78^ On the other hand, Y-STR mutations can occur at a higher rate compared to mutations in other genetic markers, which can result in rapid changes in Y-STR haplotypes over time. These high mutation rate of Y-STR markers may also contribute to their variability.^79^

 It must be noted that high mutation rate in Y-STR loci can also induce problems in interpreting the results of genetic profiles and create a major limitation, particularly when comparing profiles across different groups of a population or time period.^80^ Because the high mutation rate results in different haplotypes arising from a common ancestor, they can complicate the analysis and interpretation of genetic data and limit the investigation of possible substructures and comparison of genetic profiles.^81^ Additionally, Y-STR loci are prone to high mutation rates, and reduce the accuracy of genetic profiling.^82^ According to previous research, the high mutation rate complicates establishing the time of the most recent common ancestor (TMRCA) in genealogical applications. It may also have an effect on the variability of the human Y-chromosome and, as a result, on its application in forensic sciences, genetic genealogy, human population genetics, and molecular anthropology.^83,84^

 Populations from regions that included Iranian samples were previously analyzed using the Yfiler® marker panel.^49,51,52^ Their results showed that there was little genetic distance between socio-geographical populations in Iran. However, there was a relatively subtle substructure among groups when populations were grouped by geography. Parallel to the present study, the authors concluded that insignificant distances exist between the bulk of Iranian samples. By analyzing the patterns of Y-STR variation, they demonstrated that Iranian samples have negligible genetic differences, which may suggest that they have diverged from a common ancestor a long time ago and experienced a variety of migratory events. This variation among different ethnic groups is defined by different factors, including both geographic and linguistic ones.^21^ It is important to note that for further research, it will be beneficial to confirm our observations with a larger sample size along with assessment of mutation rates and TMRCA ensuring a comprehensive understanding of the genetic landscape in the specified area.

## Conclusion

 Genetic ancestry, partially mirrored by ethnic group affiliation, is an important factor to consider in medical research, as differences in genetic background can impact disease prevalence and response to treatments. Based on our results, we predict that among the eleven Iranian ethnic groups studied, there is no significant difference in the prevalence average in phenotypes that are influence by genetic variants on the Y-chromosome. Moreover, regarding allele frequency, these conditions can be influenced by the frequency of specific alleles in the population. By characterizing the genetic makeup of the Iranian male population, our study provides a valuable resource for future medical research and population genetics studies. By doing so, we can improve our ability to predict and prevent diseases and may develop more personalized treatment options for patients based on their genetic background and ethnic identity.
